# Management of Penile Anatomical Deformity Associated With Lymphedema: A Case Report and Literature Review

**DOI:** 10.7759/cureus.97142

**Published:** 2025-11-18

**Authors:** Periklis E Tsitsopoulos, Margarita G Toumanidou, Antonios Profka, Vasileios G Patriarcheas, Georgios Tsamos, Theodoros Mariolis-Sapsakos, Theodore Troupis, Andreas Koumenis, Dimitrios Filippou, Evangelos Dimakakos

**Affiliations:** 1 Center of Prevention, Diagnosis and Treatment of Lymphedema - Lymphatic Diseases for Adults and Children, Metropolitan Hospital, Athens, GRC; 2 First Propedeutic Department of Internal Medicine, American Hellenic Educational Progressive Association (AHEPA) University Hospital of Thessaloniki, Thessaloniki, GRC; 3 Department of Internal Medicine, Agios Dimitrios General Hospital of Thessaloniki, Thessaloniki, GRC; 4 Department of Anatomy, National and Kapodistrian University of Athens, Athens, GRC; 5 Department of Surgery, General Oncological Hospital of Kifissia Agioi Anargyroi, Athens, GRC; 6 Laboratory of Anatomy, Advanced Anatomical Applications, Artificial Intelligence and Experimental Surgical Research, National and Kapodistrian University of Athens, Athens, GRC; 7 Department of Surgery, University of Athens Medical School, Athens, GRC; 8 Department of Anatomy, University of West Attica, Athens, GRC; 9 Vascular Unit, University of Athens of Hospital Sotiria, Athens, GRC

**Keywords:** anatomy, complete decongestive therapy, lymphedema, penis lymphedema, prostatectomy, surgical treatment

## Abstract

Genital lymphedema is a well-documented, albeit uncommon, complication that may arise following prostatectomy for prostate cancer. This condition can lead to significant functional impairment and adversely affect multiple aspects of health, including daily activities, quality of life, and psychological well-being. In the present report, we describe a rare case of isolated penile lymphedema in a 59-year-old male patient following prostate surgery. This case underscores the critical role of multidisciplinary collaboration in the management of complex lymphedema presentations and highlights the therapeutic value of integrating conservative and surgical interventions. Such a combined approach can achieve not only effective reduction of lymphedema but also substantial improvements in the patient’s psychological status and overall quality of life.

## Introduction

Prostate cancer represents the second most prevalent malignancy among men worldwide, with the majority of affected patients undergoing radical prostatectomy and, in high-risk cases, concurrent lymph node dissection [1,2]. A clinically significant complication of this surgical approach, and the focus of the present case report, is the development of lymphedema.

Lymphedema is defined as a pathological swelling caused by the accumulation of lymphatic fluid in different body regions due to impaired lymphatic drainage [3,4]. It is broadly categorized into two types, namely, primary lymphedema, which occurs as a result of congenital or hereditary abnormalities of the lymphatic system, and secondary lymphedema, which develops following lymphatic vessel dysfunction or the surgical excision of lymph nodes.

The clinical course of lymphedema is commonly staged into the following four distinct phases: (a) the latent or subclinical stage, in which lymphatic impairment is present but swelling is not yet evident; (b) stage I, characterized by mild fluid accumulation that may reduce with limb elevation; (c) stage II, associated with persistent, moderate-to-severe lymphedema and frequent dermatologic changes; and (d) stage III, representing advanced, chronic lymphedema (elephantiasis) with severe disfigurement and functional compromise [4].

Lymphedema following prostate cancer surgery typically develops in the lower extremities and the genital region as a consequence of pelvic lymph node dissection. While both sites may be affected, epidemiological data demonstrate that the incidence of genital lymphedema is considerably lower than that of lower limb involvement. Specifically, reported rates range from 0-1% for genital lymphedema and 0-14% for lower limb lymphedema, increasing to 2-22% and 18-29%, respectively, in patients receiving adjuvant radiotherapy [5]. Within the genital region, lymphatic fluid accumulation occurs predominantly in the scrotum, whereas penile lymphedema remains a distinctly rare presentation.

Lymphedema contributes to substantial morbidity, affecting not only physical health but also daily activities, social participation, and overall quality of life [6]. In men, genital lymphedema after prostatectomy carries additional psychosocial consequences, with adverse effects on psychological health, sexual function, and intimate relationships, frequently contributing to psychosexual morbidity and even depressive symptoms [7].

The present case report describes, to the best of our knowledge, the first documented instance of isolated penile lymphedema following prostatectomy. The objective of presenting this case is to underscore how specialized knowledge of the patient’s lymphatic status, combined with multidisciplinary collaboration among professionals experienced in lymphedema management, can yield effective, durable outcomes in the management of such rare and complex clinical scenarios.

## Case presentation

A 59-year-old male was diagnosed with prostate cancer, presenting with an elevated prostate-specific antigen (PSA) level of 7.9 ng/mL. He subsequently underwent radical prostatectomy at Metropolitan Hospital in Athens, during which 27 lymph nodes were excised. The patient did not receive any adjuvant therapy, including chemotherapy or radiotherapy.

**Figure 1:**
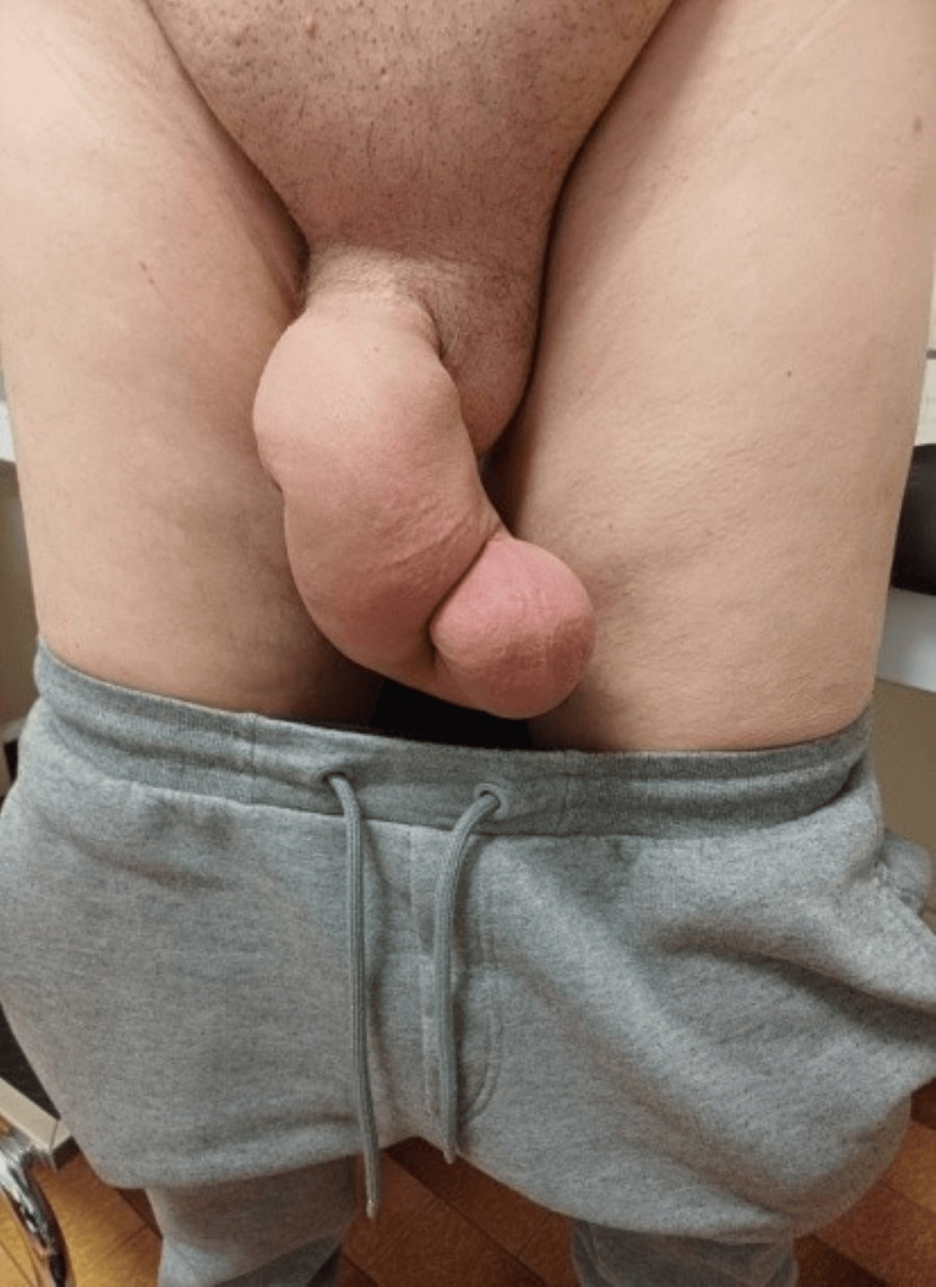
Isolated swelling of the penile shaft, without scrotal or lower limb involvement.

Six months postoperatively, the patient developed pronounced lymphatic edema localized exclusively to the penile shaft, without involvement of the scrotum or lower extremities. This isolated distribution suggested that, following radical prostatectomy and extended pelvic lymph node dissection, the normal penile lymphatic and venous drainage pathways, particularly the deep and superficial networks connecting to pelvic and inguinal nodes, were likely disrupted or rerouted, allowing retrograde or collateral flow that can, in rare cases, lead to isolated penile involvement (Figure 1). Upon referral to our center, written informed consent was obtained from the patient. Clinical examination confirmed penile lymphedema accompanied by papillomatous lesions on and around the penis. The patient was subsequently referred to a dermatologist for comprehensive evaluation and management. The dermatologist confirmed the diagnosis of human papillomavirus (HPV) infection.

Methods

Following an interdisciplinary discussion involving all specialists engaged in the management of this case, a comprehensive, phased treatment plan was implemented. The management strategy comprised three sequential phases.

Phase 1

**Figure 2:**
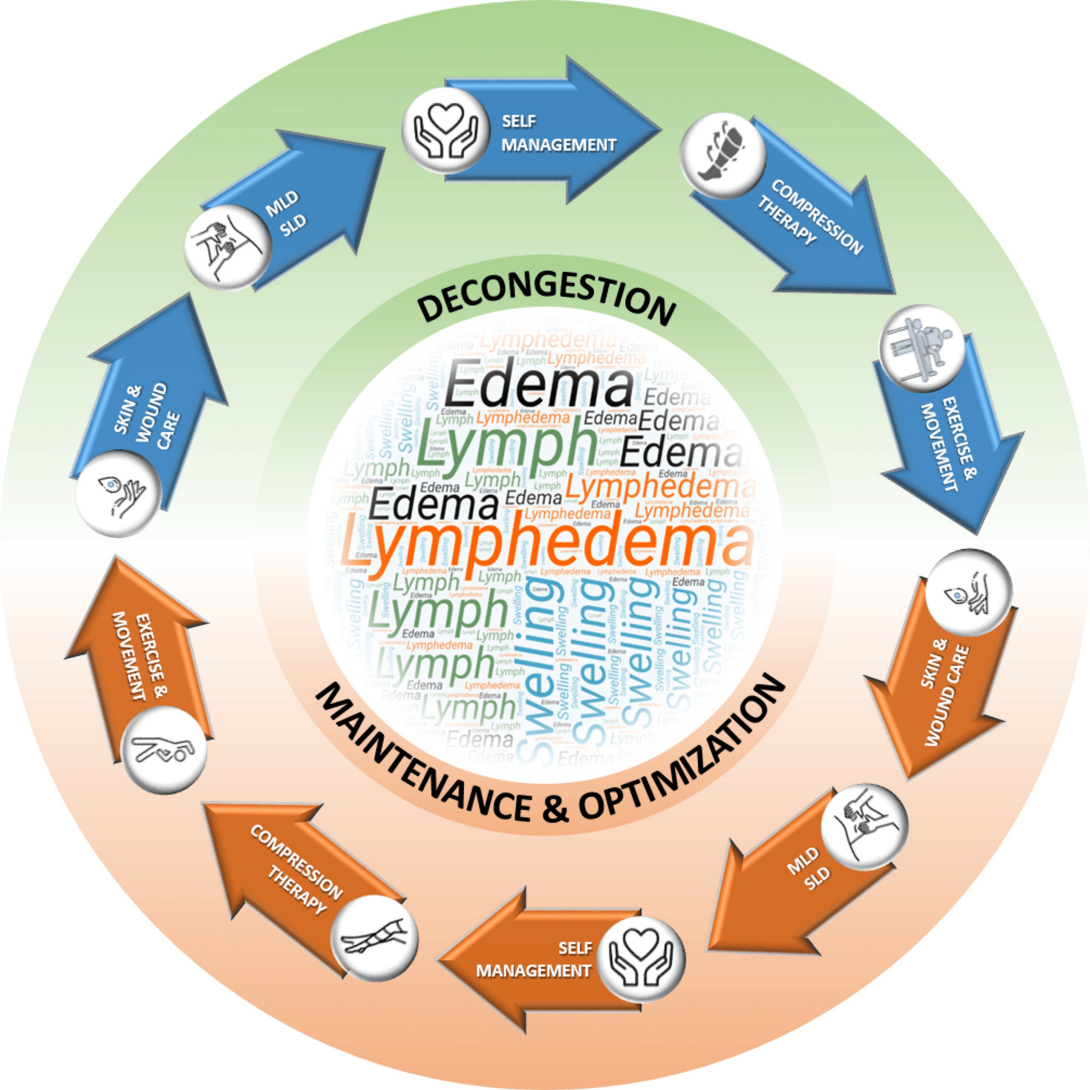
Depiction of the stages of the complete decongestive therapy technique. The image was created by the authors. MLD = manual lymphatic drainage; SLD = self-lymphatic drainage

Lasting four weeks, this initial phase addressed the concomitant HPV infection through microsurgical intervention and CO₂ laser ablation, in conjunction with conservative therapy employing complete decongestive therapy (CDT). CDT is a structured, two-stage therapeutic approach (Figure 2). During this phase, notable improvements were observed in the skin quality of the penile region, along with a significant reduction in lymphatic fluid accumulation.

Results

Phase 1: Conservative Treatment (Weeks 1-4)

**Figure 3:**
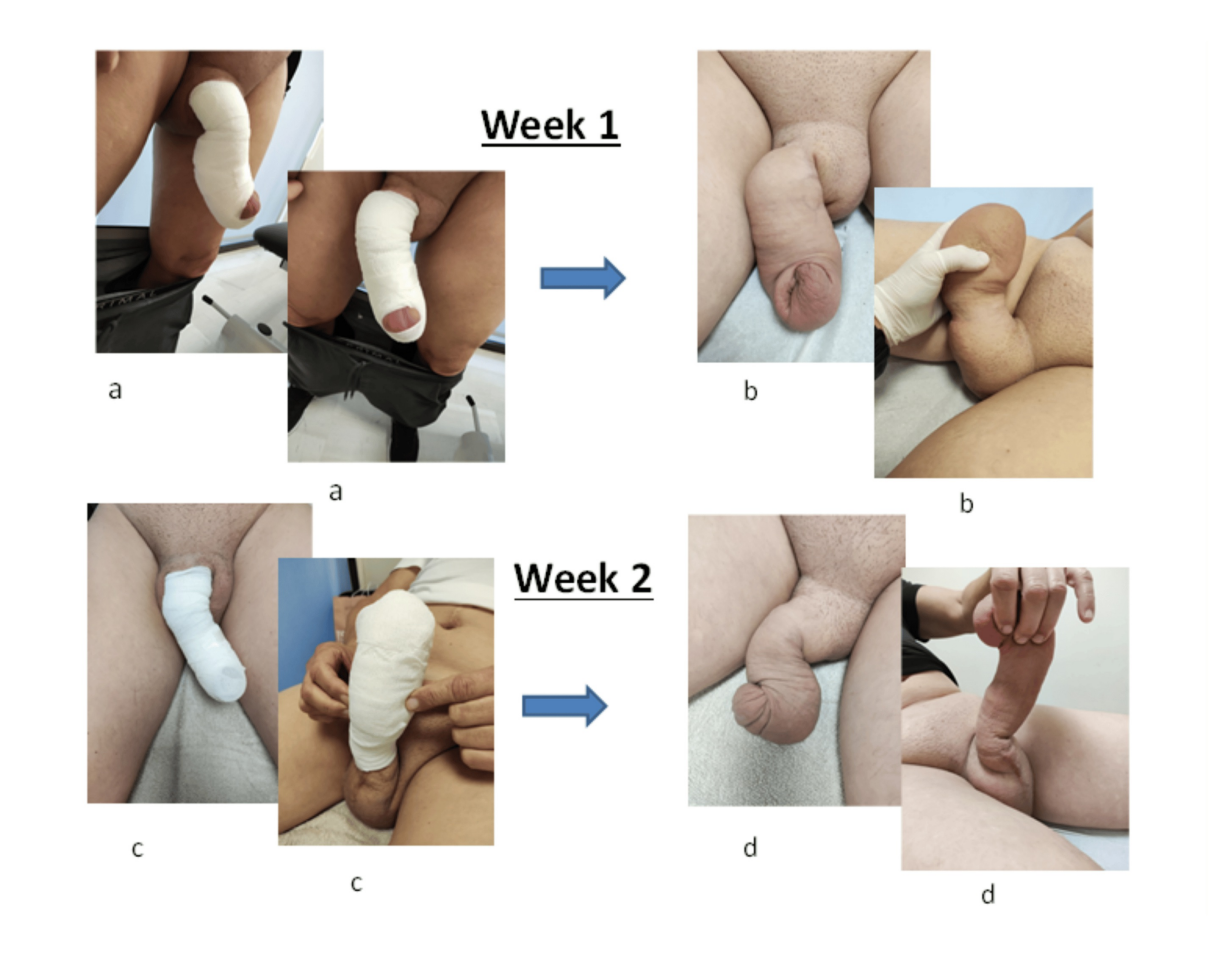
Measurement results from weeks one to two. (a) Penile compressive therapy during the first week. (b) Outcome of the therapy during the same week. (c) Penile compressive therapy during the second week. (d) Outcome of the therapy during the second week. Image illustrated by the authors. Patient consent was obtained.

**Figure 4:**
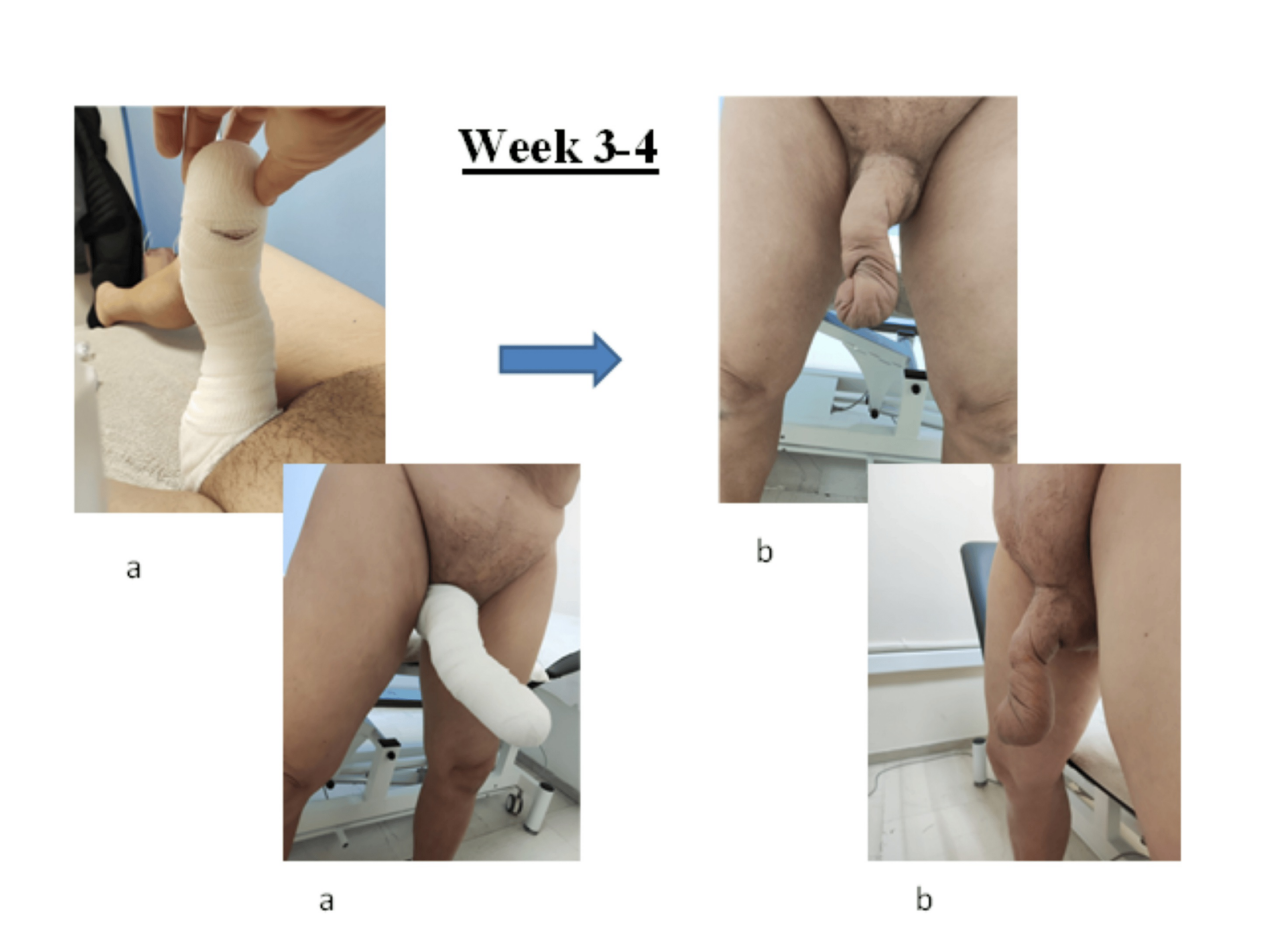
Treatment outcomes during weeks three to four. (a) Penile compressive therapy during weeks three to four. (b) Outcome of the therapy during weeks three to four. Image illustrated by the authors. Patient consent was obtained.

At the time of presentation to our center for initiation of therapy, all relevant clinical data were collected to establish a comprehensive patient profile. Baseline volumetric assessment of the affected region was performed using validated automated software (Limb Volume Professional, Bioscience Research Institute). Subsequent measurements were obtained at weeks two, three, and four to monitor progressive changes in penile volume (Figures 3, 4), and these values were systematically compared to the baseline data (Table 1).

**Table 1:** Patient’s penile circumference measurements during the first phase of therapy, as well as subsequent changes observed following the implementation of conservative complete decongestive therapy from weeks one to four. Points A-E indicate predefined locations along the penile shaft, from the base (Point A) to the tip (Point E). The numbers in parentheses in the first column represent the distance (in cm) from the penile base at which each measurement was performed.

Penile circumference measurements (21 cm shaft length)	Week 1	Week 2	Week 3	Week 4
Point A (0 cm)	16 cm	14 cm	13.5 cm	13 cm
Point B (5 cm)	19 cm	17 cm	15.5 cm	14 cm
Point C (10 cm)	23 cm	18 cm	16 cm	14 cm
Point D (15 cm)	25 cm	19 cm	16.5 cm	15 cm
Point E (20 cm)	22 cm	19 cm	17 cm	15 cm

Phase 2: Surgical Period (Weeks 5-6)

**Figure 5:**
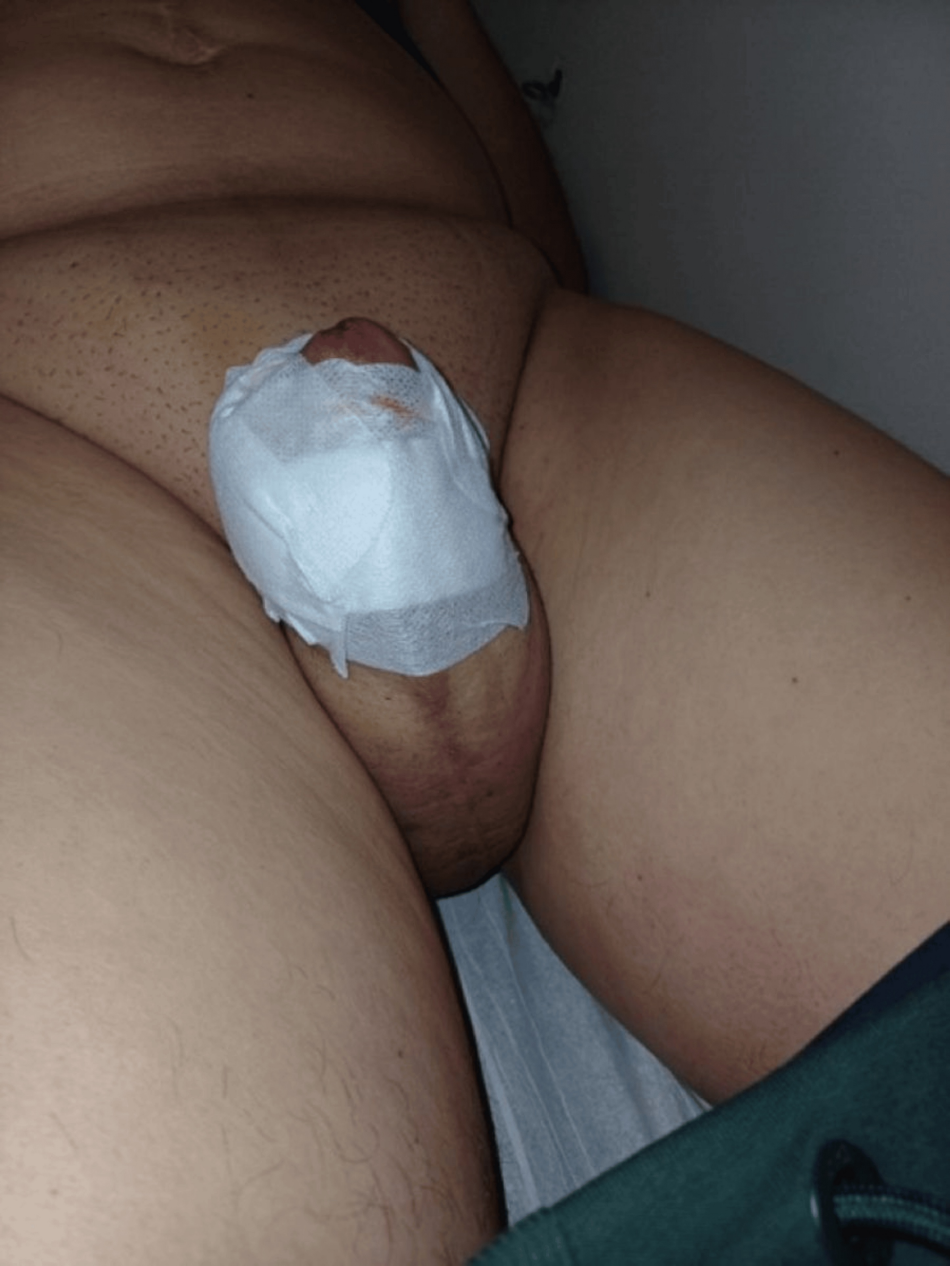
Postoperative stage during weeks five to six.

During the second phase of rehabilitation, the patient underwent surgical management of penile lymphedema, during which the majority of the excess skin was removed (Figure 5). This resulted in a substantial reduction in penile volume and restoration of the original measurements at points A and B (Table 2).

**Table 2:** Patient’s penile circumference measurements during the two-week postoperative period following surgical intervention of the penis. Points A-E indicate predefined locations along the penile shaft, from the base (Point A) to the tip (Point E). The numbers in parentheses in the first column represent the distance (in cm) from the penile base at which each measurement was performed. Points D and E are not included, as surgical intervention modified the penile length and these locations no longer correspond to the original measurement sites.

Penile circumference measurements	Week 5	Week 6
Point A (0 cm)	17 cm	18 cm
Point B (5 cm)	18 cm	18 cm
Point C (10 cm)	12 cm	10 cm
Points D, E (10-20 cm)	-	-

Phase 3: Postoperative Conservative Treatment (Weeks 7-9)

**Figure 6:**
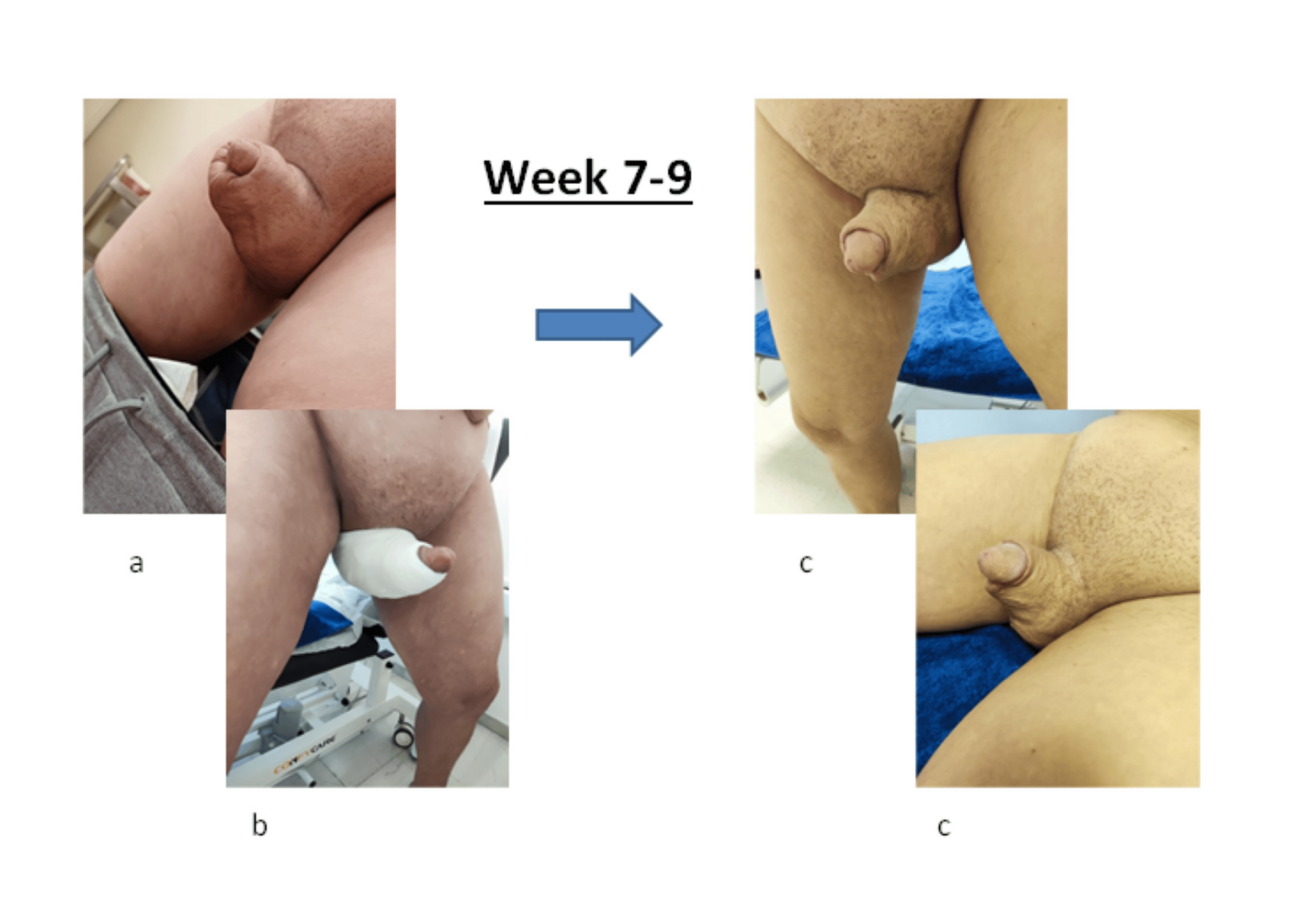
Therapeutic outcomes observed during weeks seven to nine of treatment. (a) The penis post-surgery during the seventh week, upon the patient’s return to our center. (b) The penile compressive therapy applied during weeks seven to nine. (c) Outcome of complete decongestive therapy during weeks seven to nine. Image illustrated by the authors. Patient consent was obtained.

Upon the patient’s return from surgery, conservative CDT therapy was continued to achieve complete drainage of lymphatic fluid following the surgical intervention (Figure 6). During this period, the final outcomes of the conservative treatment demonstrated a substantial improvement compared to the patient’s initial condition (Table 3).

**Table 3:** Patient’s penile circumference measurements during the three-week postoperative conservative therapy period (weeks seven to nine) using the complete decongestive therapy technique, with week nine representing the final outcome. Points A-E indicate predefined locations along the penile shaft, from the base (Point A) to the tip (Point E). The numbers in parentheses in the first column represent the distance (in cm) from the penile base at which each measurement was performed. Points D and E are not included, as surgical intervention modified the penile length and these locations no longer correspond to the original measurement sites.

Penile circumference measurements	Week 7	Week 8	Week 9
Point Α	18 cm	15.3 cm	13 cm
Point Β	18 cm	14 cm	10 cm
Point C	10 cm	9 cm	8.5 cm
Point D, E (10-20 cm)	-	-	-

Overall treatment outcomes are summarized by the changes in penile volume, measured using the validated software (Limb Volume Professional, Bioscience Research Institute), in Table 4, providing a clear quantitative representation of the improvements observed throughout the therapy.

**Table 4:** Changes in penile volume and therapy duration. Penile volume was measured using validated automated software (Limb Volume Professional, Bioscience Research Institute). Therapy duration (weeks) indicates the cumulative number of weeks of conservative complete decongestive therapy applied pre- and postoperatively.

Treatment period (weeks)	Total penile volume (mL)
Week 1	748
Week 2	498
Week 3	399
Week 4	324
Week 5	220
Week 6	214
Week 7	214
Week 8	143
Week 9	91

## Discussion

The development of lymphedema following prostatectomy represents one of the most frequently reported forms of male lymphedema. Current evidence [1,8] indicates that this complication arises predominantly in the lower extremities, typically characterized by markedly indurated skin. Importantly, a considerable subset of patients also presents with varying degrees of lymphatic fluid accumulation within the genital region. Notably, most published reports that describe lymphedema in the inguinal area refer primarily to scrotal involvement. In these cases, management strategies have been predominantly surgical [9-11], although several studies have also demonstrated the potential benefits of conservative approaches [12,13].

Beyond its physical manifestations, post-prostatectomy lymphedema is strongly associated with substantial psychosexual morbidity and an elevated risk of depression. This impact arises because genital lymphedema directly compromises multiple aspects of quality of life, including social functioning, professional activity, family relationships, and, most critically, sexual health [7]. Consequently, recognition and management of this complication are of central importance in the holistic care of prostate cancer survivors.

The clinical case presented here is, to the best of our knowledge, the first to describe a patient with prostate cancer who developed isolated penile lymphedema without scrotal involvement, and who was managed through a combination of conservative and surgical interventions. A focused review of the international literature identified only a small number of reported cases, five to six in total, of penile lymphedema secondary to chronic penile strangulation. Importantly, none of these cases were associated with prostatectomy, and all were treated exclusively with surgical intervention, without the adjunctive use of conservative therapy [14]. This underscores the uniqueness of the present case, which is distinguished not only by its clinical presentation but also by the absence of any established protocols or guidelines describing the application of CDT in such circumstances.

Particular challenges arose during the implementation of conservative CDT, especially in the bandaging phase, where standard elastic bandages could not be effectively employed due to the anatomical characteristics of the penile region. Despite these limitations, CDT remains an essential component of management. Indeed, several studies emphasize its role in optimizing surgical outcomes, as it promotes lymphatic drainage, reduces fluid accumulation, and improves skin quality, factors that are crucial in facilitating subsequent surgical intervention [13,15-17].

During surgery, a circumcision was performed, with excision of the redundant skin extending from the mid-shaft of the penis to the distal end, thereby exposing the glans. The remaining excess skin was then sutured at the coronal sulcus.

Two weeks postoperatively, the patient returned to our center for completion of the conservative phase of therapy, reporting high satisfaction with the aesthetic outcome. Over the subsequent three weeks of conservative management, difficulties related to bandaging were noticeably reduced compared with the preoperative period.

Completion of the therapeutic process resulted in a clear improvement of the patient’s clinical status, accompanied by enhanced quality of life and a significant positive impact on psychological well-being without wearing any elastic garment.

This case highlights the critical importance of interdisciplinary collaboration in both the conservative and surgical management of penile lymphedema. The integration of specialized expertise regarding the patient’s lymphatic condition, the application of CDT, and the surgical intervention produced highly favorable outcomes. These included a marked reduction of lymphatic fluid accumulation, restoration of skin quality and function, and substantial improvements in overall quality of life.

## Conclusions

Penile lymphedema represents a rare yet serious complication that may occur following prostatectomy for prostate cancer. This condition can profoundly affect both the physical health and psychological well-being of affected patients, potentially leading to deterioration in quality of life. The present case highlights the clinical relevance of structured management strategies in such challenging scenarios. In this patient, a carefully integrated therapeutic approach appeared to restore physiological function and support psychological resilience. Nevertheless, the findings are limited by the short follow-up period and the single-case nature of this report, which restrict generalizability. Future research should aim to establish standardized guidelines for penile CDT and develop imaging-based assessment criteria to enhance diagnostic accuracy and treatment evaluation.
